# Physical activity intervention for non-diabetic patients with non-alcoholic fatty liver disease: a meta-analysis of randomized controlled trials

**DOI:** 10.1186/s12876-020-01204-3

**Published:** 2020-03-12

**Authors:** Shu-ting Wang, Jing Zheng, He-wei Peng, Xiao-lin Cai, Xin-ting Pan, Hui-quan Li, Qi-zhu Hong, Xian-E Peng

**Affiliations:** 1 Department of Epidemiology and Health Statistics, Fujian Provincial Key Laboratory of Environment Factors and Cancer, School of Public Health, Fujian Medical University, Fuzhou, 350122 China; 2 Key Laboratory of Ministry of Education for Gastrointestinal Cancer, Fujian Medical University, Fujian, China

**Keywords:** Physical activity, Nonalcoholic fatty liver disease, Randomized controlled trials; meta-analysis

## Abstract

**Background:**

Non-alcoholic fatty liver disease (NAFLD) is currently the most common cause of chronic liver disease nowadays. Changes in diet and lifestyle have led to a dramatic increase in the prevalence of NAFLD around the world. This meta-analysis is to investigate the efficacy of physical activity intervention on liver-specific endpoints in the population with NAFLD, including hepatic enzyme, serum lipid, glucose metabolism and intra-hepatic lipid.

**Methods:**

PubMed and China National Knowledge Infrastructure (CNKI) databases were searched for randomized clinical trials of physical activity intervention on NAFLD patients through April 20th, 2019. Effect sizes were reported as standardized mean difference (SMD) and 95% confidence intervals (CI). Quality of included studies was assessed according to the Cochrane risk of bias tool. Meta-analyses were conducted using random-effect or fixed-effect models depending on the significance of heterogeneity. Subgroup analyses according to types and duration of physical activity were conducted to investigate clinical variability.

**Results:**

Nine studies with a cumulative total of 951 participants met selection criteria. Physical activity was found associated with small reductions in hepatic enzyme parameters: ALT (SMD -0.17, 95% CI:-0.30 to − 0.05), AST (SMD -0.25, 95% CI: − 0.38, − 0.13) and GGT (SMD -0.22, 95% CI: − 0.36, − 0.08). Significant small improvements were also found in serum lipid parameters including TC (SMD = − 0.22, 95% CI: − 0.34, − 0.09), TG (SMD = − 0.18, 95% CI: − 0.31 to − 0.06) and LDL-C (SMD = − 0.26, 95% CI: − 0.39 to − 0.13). Significant improvement was also found in intra-hepatic lipid content (SMD = − 0.21, 95% CI: − 0.36 to − 0.06) There was no difference between physical intervention group and control group in HDL and three glucose metabolism parameters. Subgroup analysis suggested both aerobic exercise alone and resistance exercise alone can improve most liver function and longer period of exercise generally had better improvement effect.

**Conclusions:**

Our findings suggest that physical activity alone can only slightly improve hepatic enzyme levels, most serum lipid levels and intra-hepatic lipid content in non-diabetic patients with NAFLD.

## Background

Non-alcoholic fatty liver disease (NAFLD) is a multi-system disease characterized with fat storage and hepatic steatosis not caused by excessive drinking. NAFLD encompasses a wide histological spectrum ranging from simple steatosis to non-alcoholic steatohepatitis (NASH), liver fibrosis and cirrhosis, which can result in liver cancer. Nowadays, dramatic changes in the lifestyle and diet of the global population are causing the prevalence of NAFLD increasing rapidly, in parallel with that of obesity and diabetes [1]. NAFLD is estimated to replace viral hepatitis as the primary factor for end-stage liver disease and liver transplantation by 2023 [2]. Globally, the prevalence of NAFLD varies among different countries and regions. Most Asian countries and developing countries have the low prevalence of NAFLD (about 10%), while China and Japan, as well as the United States, Europe, Middle-East, Latin America and Australia, have the high prevalence of NAFLD ranges from 20 to 30% [3]. Additionally, based on the result of one meta-analysis from mainland China, the prevalence of NAFLD in China is reaching 45% [4]. NAFLD is becoming one of the most important public health issues around the world. The first-line management for NAFLD is lifestyle modification, including dietary restriction and increased habitual physical activity [5]. Physical activity as a lifestyle modification plays an important role in the development of NAFLD. Previous studies found an inverse relationship between physical activity and the risk of NAFLD [6, 7]. However, clinical trials examining the therapeutic benefit of physical activity in NAFLD have reported inconsistent results [8, 9], and the effectiveness and biological mechanism of physical activity, independent of diet, remain insufficient. NAFLD is strongly associated with features of the metabolic syndrome, especially type 2 diabetes mellitus (T2DM), as over 70% of patients with T2DM have NAFLD [10]. However, the causality between NAFLD and T2DM is not entirely understood. Our objective, therefore, was to conduct a meta-analysis of the pooled data from published randomized controlled trails (RCTs) to evaluate the effectiveness of physical activity intervention on hepatic enzyme, serum lipid metabolism, glucose metabolism and intra-hepatic lipids content in non-diabetic patients with NAFLD [11], which will provide substantial evidence on whether physical activity intervention has a favorable effect for treating NAFLD.

## Methods

### Data sources and search strategy

We performed a comprehensive search for English and Chinese language publications on PubMed and China National Knowledge Infrastructure (CNKI) databases through April 20th, 2019. The search terms used were physical activity, exercise, aerobic exercise, aerobic training, resistance exercise, resistance training, strength exercise, strengthening exercise, strength training, NAFLD, NASH, NAFL, non-alcoholic fatty liver disease, non-alcoholic steatohepatitis, fatty liver, liver steatosis and hepatic steatosis. The reference lists cited in the selected articles and published reviews were manually searched to identify additional studies.

### Inclusion and exclusion criteria

Two reviewers (Shu-Ting Wang and Jing Zheng) independently screened the literature selected from the initial search. Studies were included if they fulfilled the following inclusion criteria: (1) prospective randomized controlled trials; (2) population of any age or sex or ethnic origin with NAFLD diagnosed based on the standard guidelines using noninvasive or invasive approaches; (3) intervention involving any type of physical activity with any intensity level or duration; (4) comparison with placebo (sham exercise) or usual care without physical activity; (5) outcomes of interest were improvements in hepatic enzyme level, serum lipid level, glucose metabolism and intra-hepatic lipids content.

The exclusion criteria were: (1) only abstract available; (2) non-human study; (3) studies enrolled populations with secondary causes of fatty liver such as alcohol, hepatitis viruses or medication; (4) studies included patients with other metabolic issues, like diabetes.

### Data extraction and quality assessment

A pre-designed data collection form was used to extract the following information: first author, year of publication, country, trial’s design type, number of enrolled populations, participants’ character, intervention’s character, duration of follow-up and method for NAFLD diagnosis. If the articles contained insufficient information, we contacted the authors to obtain the missing details.

The methodological quality of each included study was assessed using the Cochrane risk of bias tool in Review Manager 5.3 (Nordic Cochrane Centre, Copenhagen, Denmark), which includes the following items: randomized sequence generation; allocation concealment; blinding of participants and personnel; blinding of outcome assessment; incomplete outcome data; selective reporting and other bias. The studies were classified into risk of low, unclear, or high bias risk.

Two reviewers performed all data extraction and quality assessment independently. If discrepancies occurred, a consensus result was achieved by discussion.

### Statistical analysis

The outcomes in this study are all continuous variables presented as standardized mean difference (SMD) and 95% confidence intervals (CI). Heterogeneity was evaluated using the Cochran Q statistic and I^2^ metric. A random-effect model was used to pool the study data if I^2^ value > 50% which represented statistical heterogeneity; otherwise, a fixed-effect model was used. Because it is difficult to interpret the clinical effect size of SMD [12], we used the interpretation thresholds proposed by Cohen et al. who suggested SMDs < 0.2, ≥0.2 and < 0.5, ≥0.5 and < 0.8, ≥0.8 correspond to insignificant, small, moderate and large effect sizes respectively [13]. To investigate clinical variability, subgroup analyses based on the type and duration of physical activity was conducted. Sensitivity analyses were further performed by removing each study individually to evaluate the stability and reliability of the results of the primary meta-analysis. When there were at least 10 studies included in the meta-analysis, publication bias was detected by Egger’s regression test, with *p* < 0.05 indicative of significance. All the above statistical analyses were conducted using R software (Version 3.5.3).

## Results

### Literature search and study characteristics

**Fig. 1:**
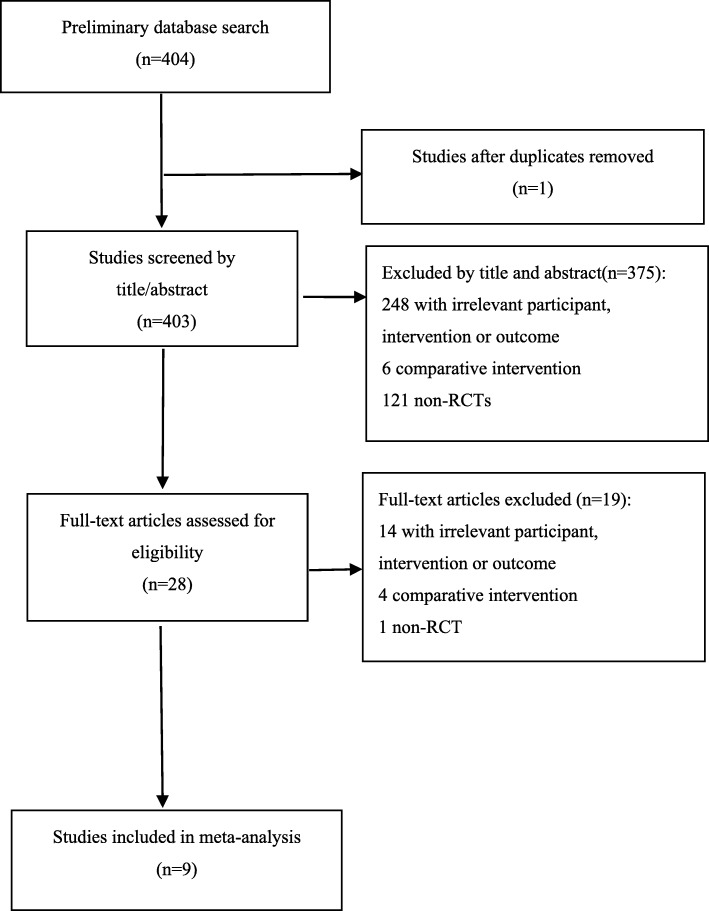
Flowchart of the study selection process

Figure 1 shows the details of the literature search and study selection. Our search strategy initially identified 404 papers. Duplicate removal and screening through article title and abstract review identified 28 studies. Nineteen studies were excluded after the full text was reviewed. Finally, 9 studies involving 951 participants were included in the meta-analysis [8, 14–21]. Among them, 2 studies [17, 21] had more than one intervention groups, therefore each type of intervention was compared with control group and analyzed. Table 1 outlines the characteristics of the included studies. The majority of included studies reported both male and female NAFLD patients except one involving only male patients [19]. All studies involved adults NAFLD patients. The intervention duration ranged from 8 to 48 weeks and the median was 16 weeks (4 months).

**Table 1:** Characteristics of the included studies

Study	Design	Enrolled (n)	Participants	Intervention characteristics			Control	Method for NAFLD diagnosis
				Physical activity type	Sessions per week	Intervention duration (weeks)		
Sullivan 2012 [14]	monocentre	18	Obese adults with NAFLD	Aerobic	5 times	16	Continue current activities of daily living	1H MRS
Pugh 2013 [8]	monocentre	13	Adults with NAFLD	Aerobic	3–5 times	16	Basic lifestyle advice	Biochemical tests
Pugh 2014 [15]	monocentre	31	Obese adults with NAFLD	Aerobic	3–5 times	16	Conventional care	MRI
Zelber-Sagi 2014 [16]	monocentre	82	Adults with NAFLD	Resistance	3 times	12	Sham exercise (stretching)	Ultrasonography
Shamsoddini 2015 [17]	monocentre	30	Male adults with NAFLD	Aerobic +Resistance	135 min	8	No intervention	Ultrasonography
Cuthbertson 2016 [18]	monocentre	69	Adults with NAFLD	Aerobic	3 times	16	Conventional counselling	MRI
Shojaee 2016 [19]	monocentre	27	Male adults with sedentary lifestyles and NAFLD	Aerobic + Resistance	4–5 times	16	Standard care	Ultrasonography or Liver biopsy
Zhang 2016 [20]	monocentre	220	Obese adults with NAFLD	Aerobic	150 min	48	No intervention	1H MRS
Jia 2018 [21]	monocentre	461	Adults with NAFLD	Aerobic + Resistance	≥3 times	24	Standard care	Ultrasonography

### Methodological quality

**Fig. 2:**
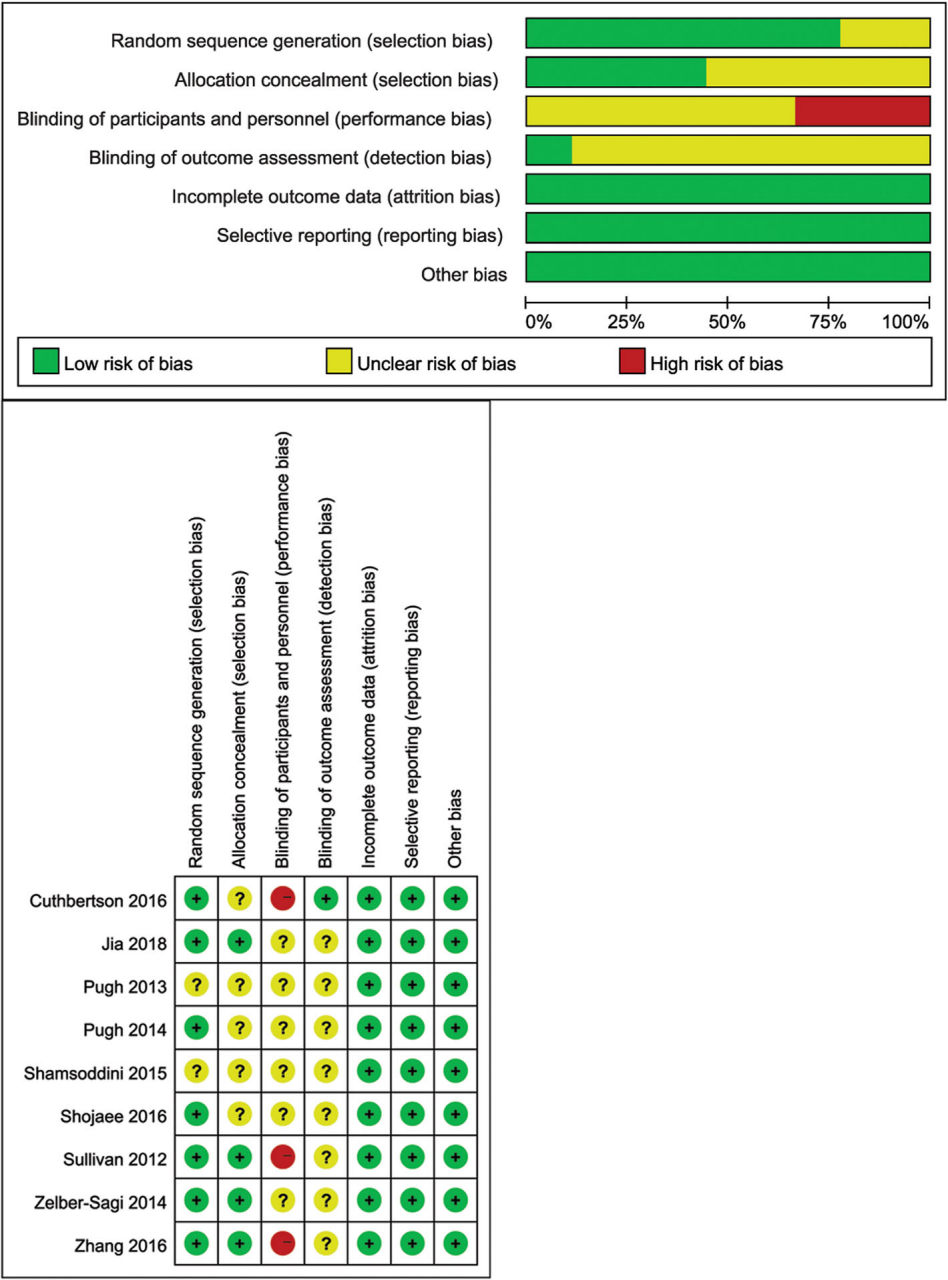
Methodological quality and risk of bias of the included trials

Figure 2 presents the methodological quality of the included studies. Most studies had low risk of bias in random sequence generation and all four studies reported information in allocation concealment had low risk of selection bias; however, three studies had high risk in blinding of participants and personnel and only one study reported the use of blinding in outcome assessment. All studies had low risk of bias in incomplete outcome data and selective reporting.

### Effect of physical activity on hepatic enzyme parameters

**Fig. 3:**
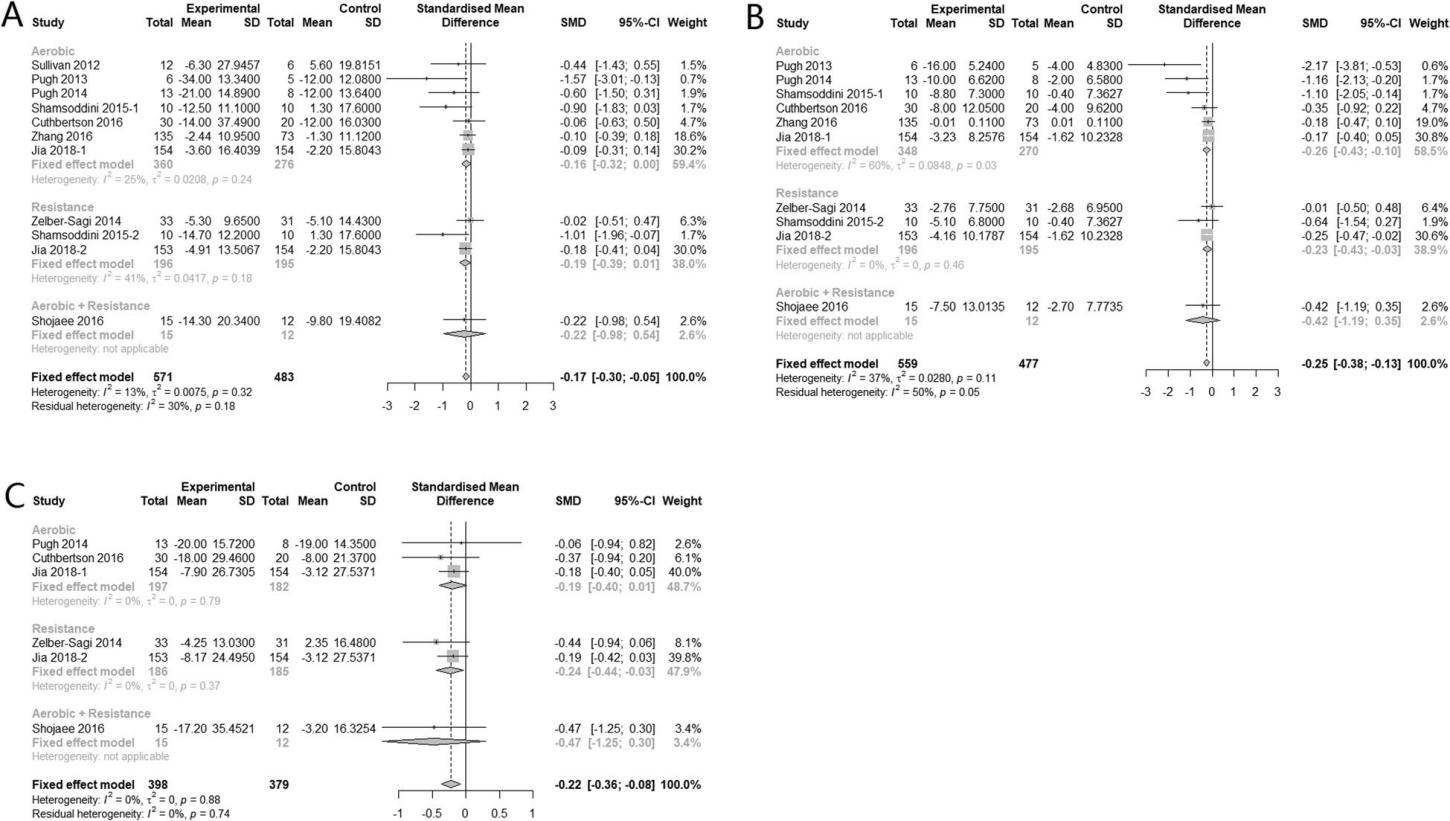
Subgroup analysis of the effects of physical activity intervention type on hepatic enzyme parameters (**a**: ALT, **b**: AST, **c**: GGT)

Nine, eight and five studies reported data for alanine aminotransferase (ALT), aspartate aminotransferase (AST), and γ-glutamyl transferase (GGT) respectively. The combined results suggested that physical activity alone can significantly improve all three hepatic enzyme parameters: ALT (SMD -0.17, 95% CI: − 0.30 to − 0.05), AST (SMD -0.25, 95% CI: − 0.38, − 0.13) and GGT (SMD -0.22, 95% CI: − 0.36, − 0.08), and the heterogeneity among studies were all insignificant (I^2^ < 50%). But the effect size is generally small with marginal confidence interval (Fig*.* 3).

Compared with no physical activity, subgroup analysis showed that aerobic exercise alone can significantly reduce AST (SMD -0.26, 95% CI: − 0.43, − 0.10). Regarding to ALT and GGT, the effects were insignificant (SMD -0.16, 95% CI: − 0.32 to 0.00 and SMD -0.19, 95% CI: − 0.40 to 0.01 respectively). Resistance exercise alone had similar small improvement effect on these two hepatic enzyme parameters: AST (SMD -0.23, 95% CI: − 0.43, − 0.03) and GGT (SMD -0.24, 95% CI: − 0.44, − 0.03). But regarding to the combination of aerobic and resistance exercise, results showed no improvements on all three parameters. Heterogeneity between subgroups were insignificant for ALT (*p* = 0.32), AST (*p* = 0.11) and GGT (*p* = 0.88) (Fig*.* 3).

**Fig. 4:**
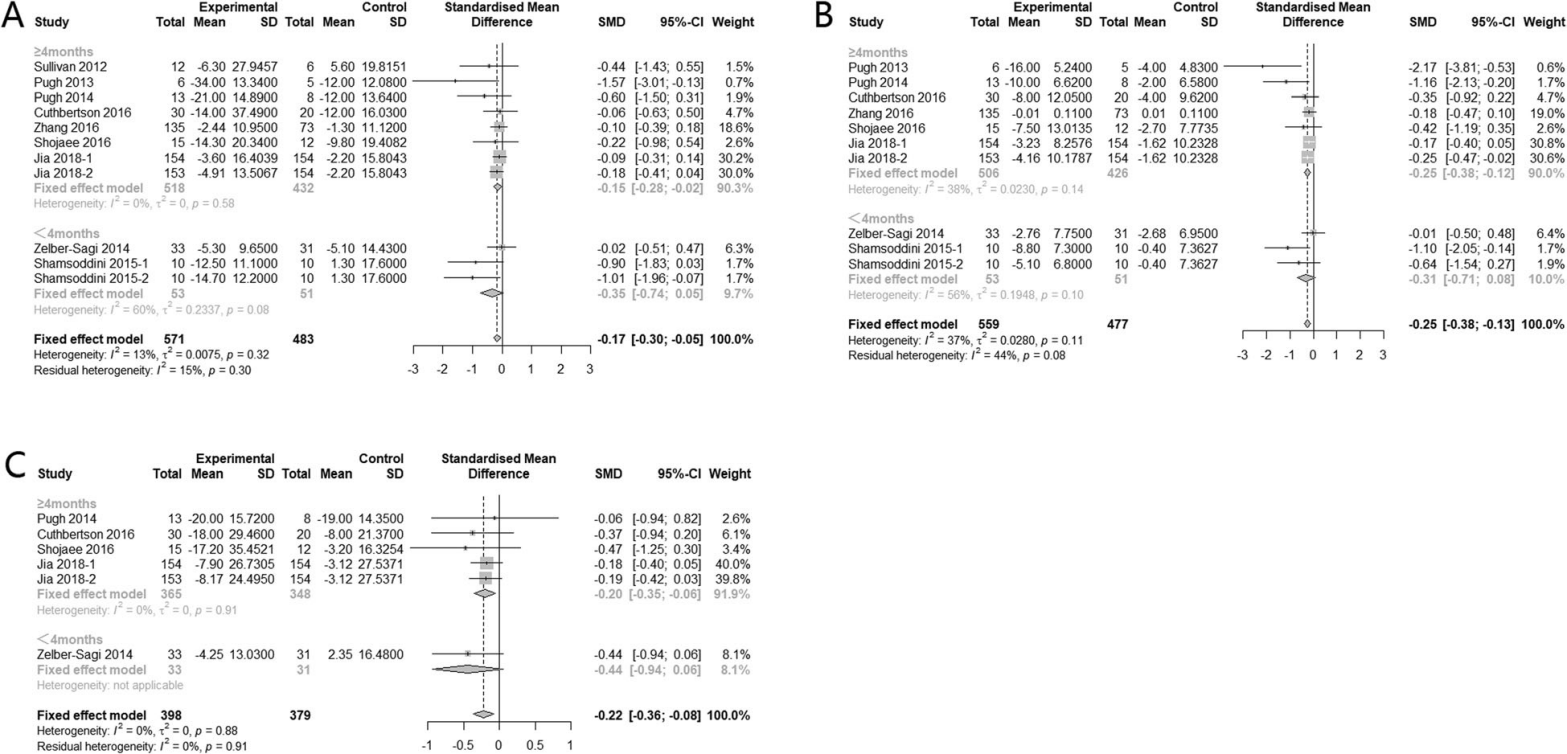
Subgroup analysis of the effects of physical activity intervention duration on hepatic enzyme parameters (**a**: ALT, **b**: AST, **c**: GGT)

Further categorizing studies according to the intervention duration, subgroup analysis showed that, regardless of the type of physical activity, keeping regular physical activity for more than 4 months can significantly improve hepatic enzyme level, while less than 4 months’ physical activity had no significant effect (Fig*.* 4).

### Effect of physical activity on serum lipid parameters

**Fig. 5:**
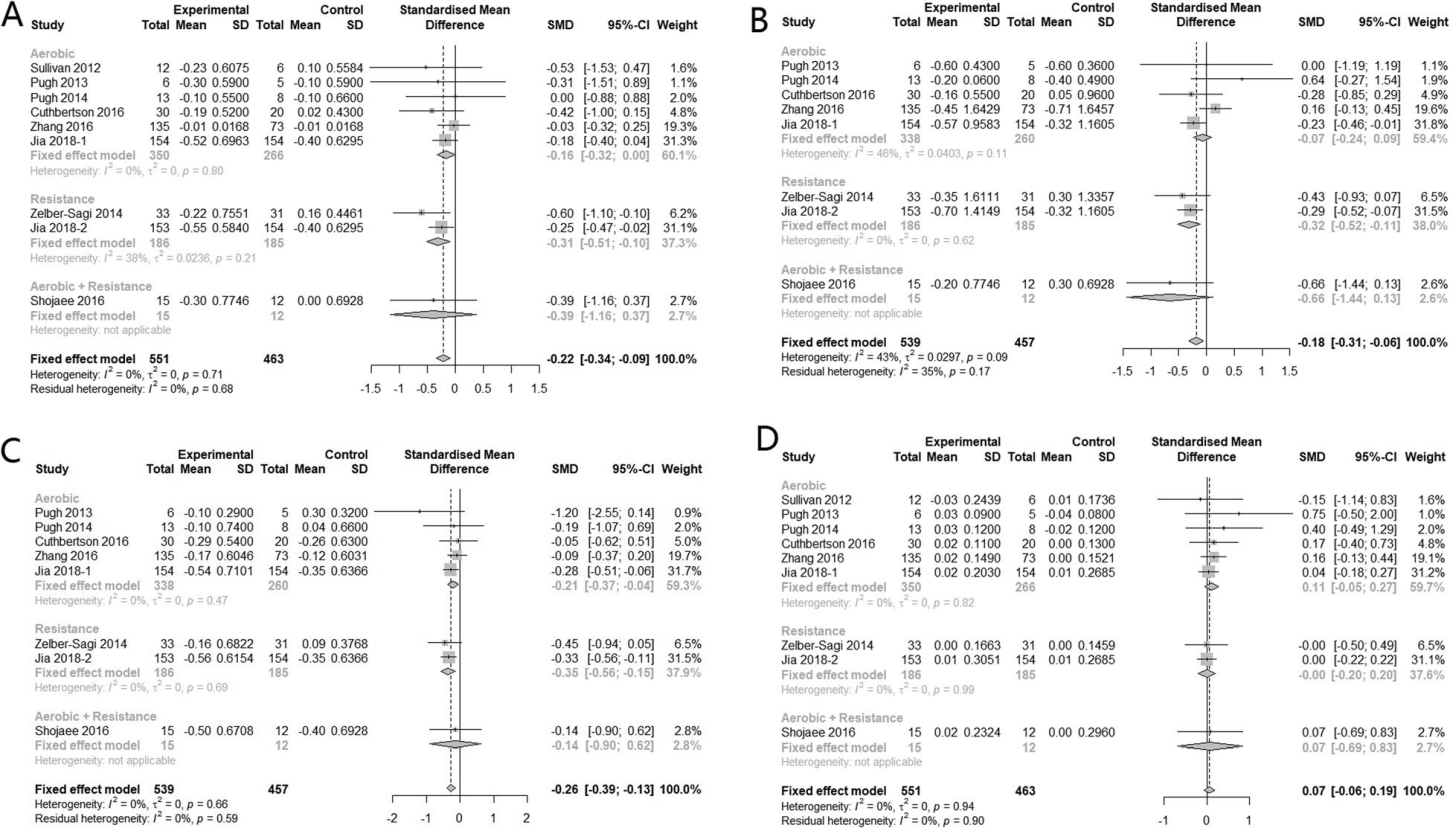
Subgroup analysis of the effects of physical activity intervention types on serum lipid parameters (**a**: TC, **b**: TG, **c**: LDL, **d**: HDL)

Eight, seven, seven, and eight studies had sufficient data for inclusion in meta analyses for total cholesterol (TC), triglycerides (TG), low-density lipoprotein cholesterol (LDL-C), and high-density lipoprotein cholesterol (HDL-C), respectively. There was no significant heterogeneity among these studies for all these four parameters (I^2^ < 50%). The pooled result showed that, compared with control group, participants who had regular physical activities were more likely to have slightly lower TC (SMD = − 0.22, 95% CI: − 0.34, − 0.09), TG (SMD = − 0.18, 95% CI: − 0.31, − 0.06) and LDL-C (SMD = − 0.26, 95% CI: − 0.39, − 0.13). Regarding to HDL-C, physical activity tended to increase the level of it compared with control, but the effect was insignificant (SMD = 0.07, 95% CI: − 0.06, 0.19) (Fig*.* 5). In the subgroup analysis, taking resistance exercise alone can significantly reduce TC but with a small effect size (SMD = − 0.31, 95% CI: − 0.51, − 0.10), while neither aerobic exercises alone nor combination of it with resistance exercise can improve TC. Heterogeneity between subgroups were not significant (*p* = 0.71). As for TG, only resistance exercise alone can significantly reduce the level of it, with small effect size (SMD = − 0.32, 95% CI: − 0.52 to − 0.11). The heterogeneity between subgroups for TG was significant (*p* = 0.09). The subgroup analyses also suggested that aerobic exercises alone and resistance exercise alone can significantly reduce the level of LDL-C, with SMD -0.21, 95% CI: − 0.37 to − 0.04 and SMD -0.35, 95% CI: − 0.56 to − 0.15 respectively, but combination of aerobic and resistance exercise had no significant effect on LDL-C, which may due to small sample size as only 1 study was included in this subgroup. The heterogeneity between subgroups was insignificant (*p* = 0.66). Regarding HDL-C, all three subgroups showed insignificant results and there was no difference between subgroups (*p* = 0.94) (Fig*.* 5).

**Fig. 6:**
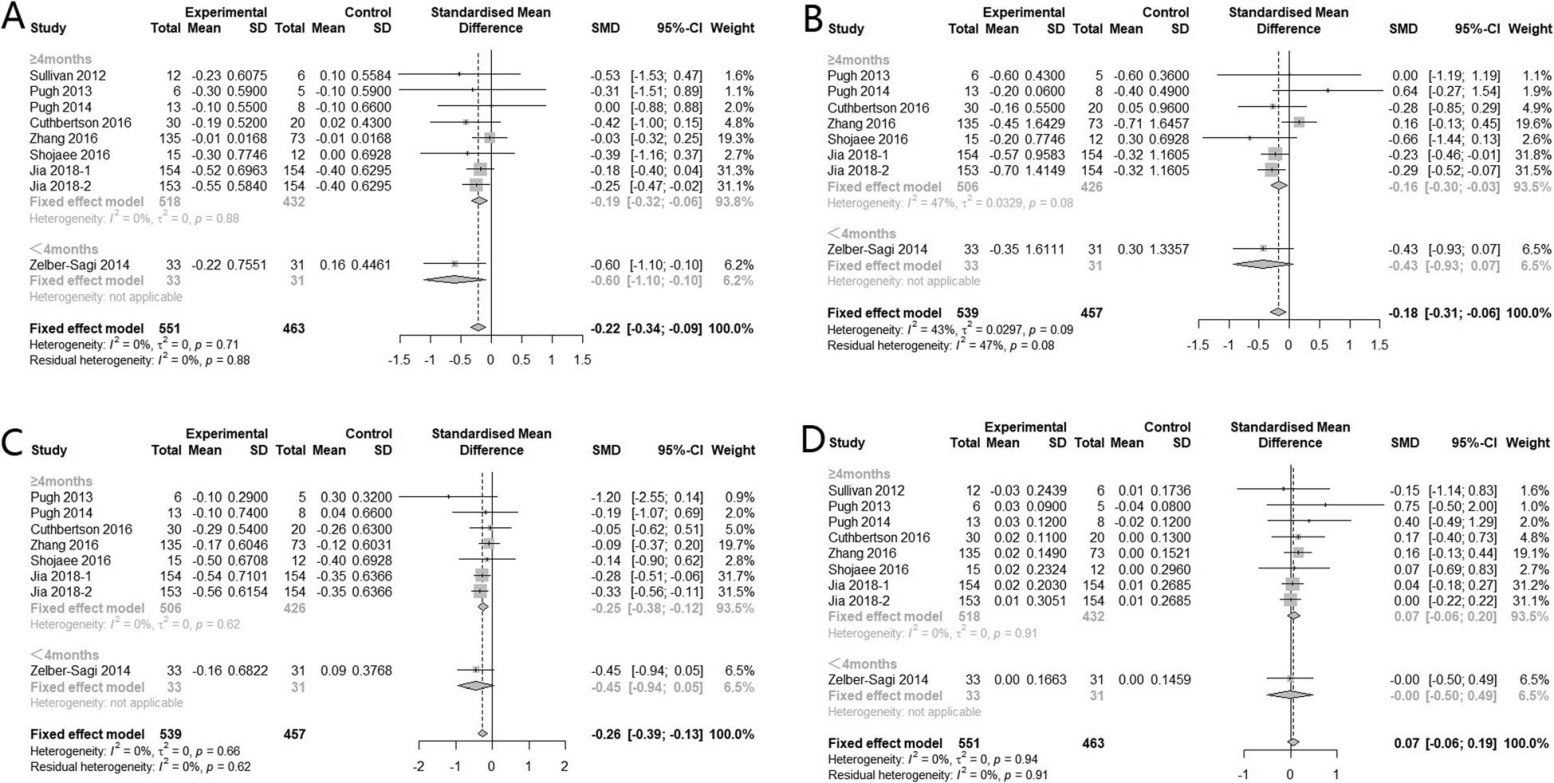
Subgroup analysis of the effects of physical activity intervention duration on serum lipid parameters (**a**: TC, **b**: TG, **c**: LDL, **d**: HDL)

As for duration of physical activity, both ≥4 months and <4 months groups had significant effect on TC regardless of the type of physical activity (SMD -0.19, 95% CI − 0.32 to − 0.06 and SMD -0.60, 95% CI − 1.10 to − 0.10 respectively). Only ≥4 months intervention significantly improved TG (SMD -0.16, 95% CI: − 0.30 to − 0.03) and LDL-C (SMD -0.25, 95% CI: − 0.38 to − 0.12). Regarding HDL-C, both subgroups showed insignificant effect (Fig*.* 6).

### Effect of physical activity on glucose metabolism parameters

Seven, four and four studies had sufficient data for inclusion in analyses of fasting glucose, fasting insulin, and homeostasis model assessment of insulin resistance (HOMA-IR), respectively. The heterogeneity among studies for these three glucose metabolism parameters was all significant (I^2^>50%). The random-effect model showed that there was no significant effect of physical activity on improving these glucose metabolism parameters.

**Fig. 7:**
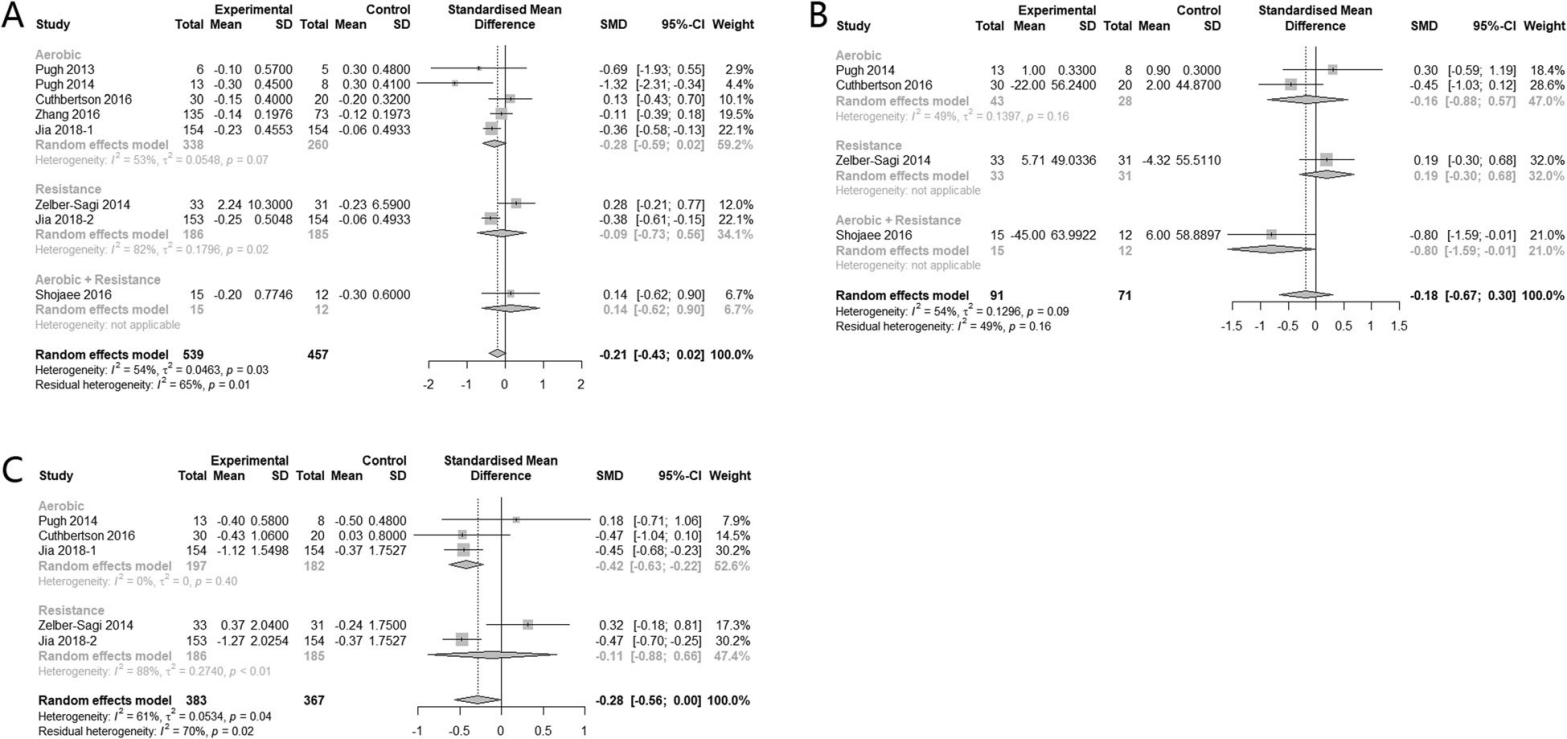
Subgroup analysis of the effects of physical activity intervention types on glucose metabolism parameters (**a**: fasting glucose, **b**: fasting insulin, **c**: HOMA-IR)

In the subgroup analysis, compared with control, only aerobic exercise alone can significantly reduce HOMA-IR (SMD = − 0.42, 95% CI: − 0.63 to − 0.22). Heterogeneities between subgroups for HOMA-IR was significant (*p* = 0.04). Combination of aerobic exercise and resistance exercise can improve fasting insulin (SMD = − 0.80, 95% CI: − 1.59 to − 0.01), but this subgroup only included 1 study with a small sample size of 15. As for fasting glucose, all three types of physical activity had no significant improvement effect (Fig*.* 7).

**Fig. 8:**
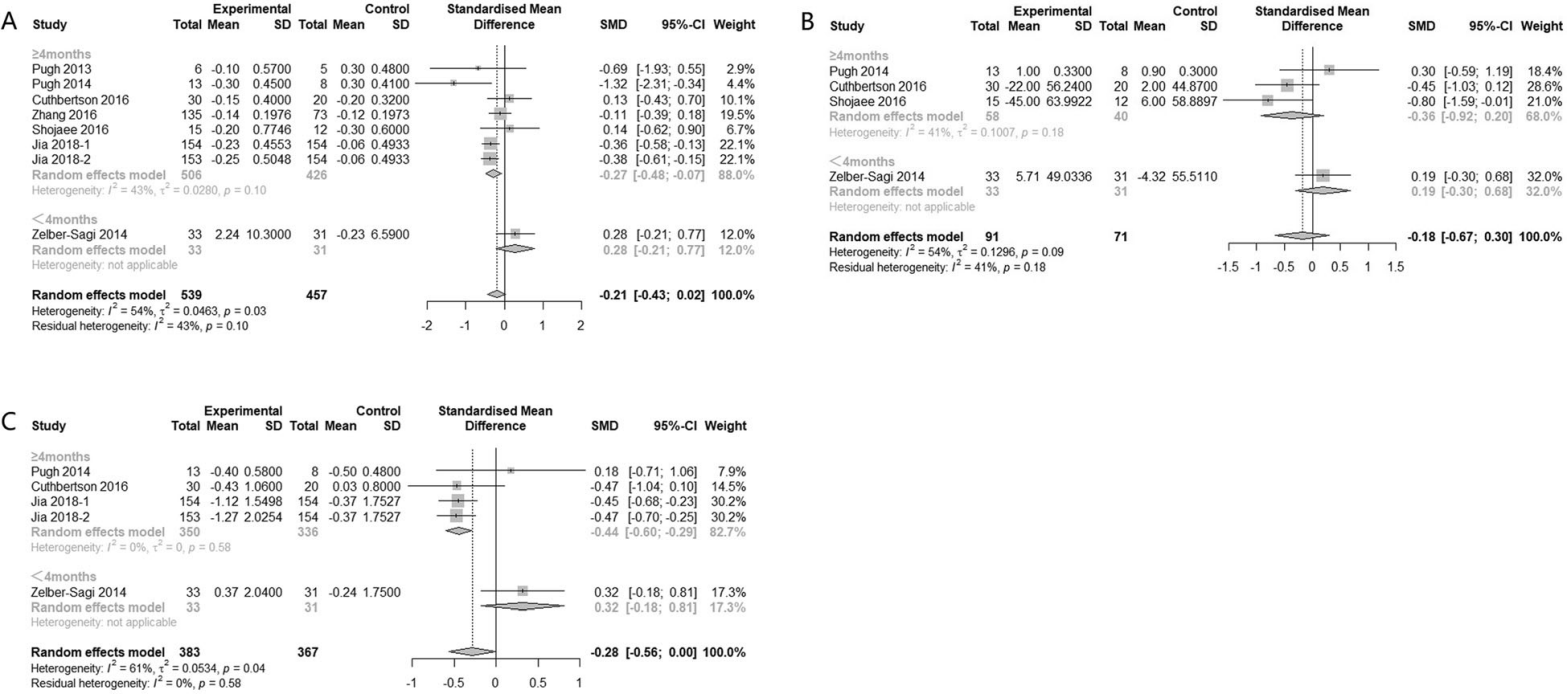
Subgroup analysis of the effects of physical activity intervention duration on glucose metabolism parameters (**a**: fasting glucose, **b**: fasting insulin, **c**: HOMA-IR)

In the subgroup analysis according to duration of intervention, significant improvement was found in ≥4 months group for fasting glucose (SMD = − 0.27, 95% CI: − 0.48, − 0.07) and HOMA-IR (SMD = − 0.44, 95% CI: − 0.60, − 0.29). There were no significant effects in subgroups for fasting insulin (Fig*.* 8).

### Effect of physical activity on intra-hepatic lipid content

**Fig. 9:**
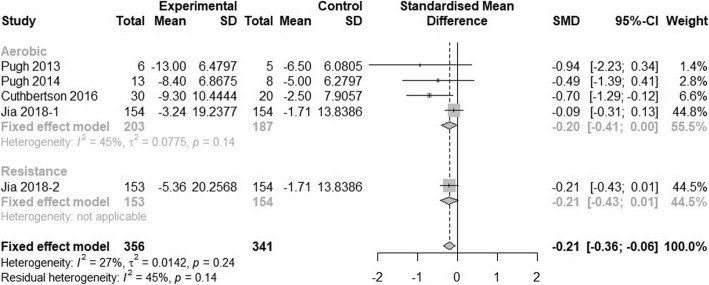
Subgroup analysis of the effect of physical activity intervention types on intra-hepatic lipid content

Four studies had sufficient data to be included in the analysis of effect of physical activity on intra-hepatic lipid content. The heterogeneity among studies was not significant (I^2^ < 50%). The fixed-effect model showed that physical activity can significantly reduce NAFLD patient’s liver fat content (SMD = − 0.21, 95% CI: − 0.36 to − 0.06). In the subgroup analysis of different intervention types, neither aerobic exercise nor resistance exercise showed significant improvement on intra-hepatic lipid content, but only one study had an intervention arm of resistance exercise with 153 participants (Fig. 9). Because all four included studies had intervention durations longer than 4 months, the effect of physical activity on intra-hepatic lipid cannot be assessed in subgroup analysis.

### Sensitivity analysis and publication bias

**Fig. 10:**
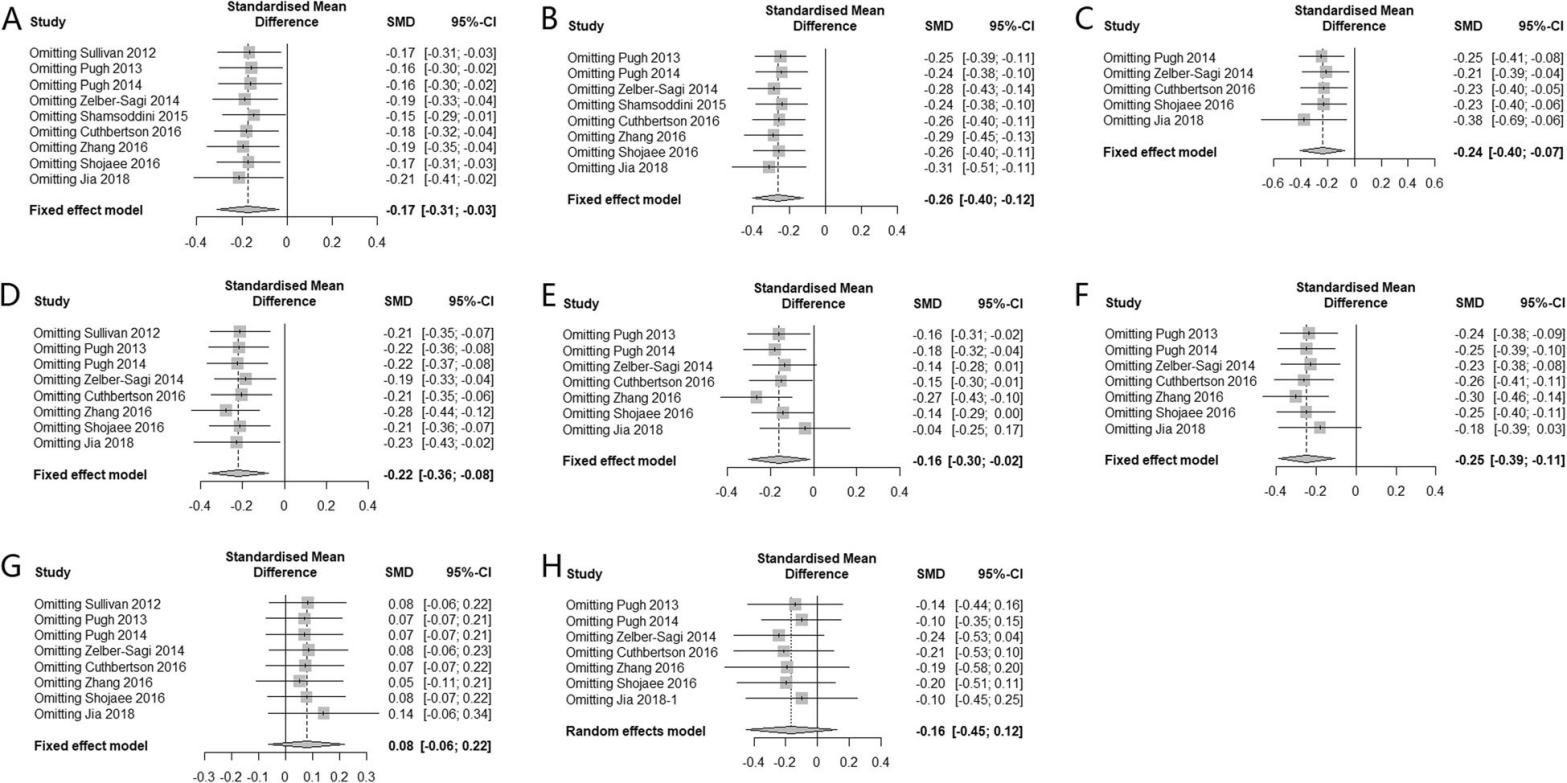
Sensitivity analysis of the effect of physical activity intervention on NAFLD

Sensitivity analysis was conducted by removing each individual study, except for fasting insulin, HOMA-IR, and intra-hepatic lipid content where only 4 studies were included. The results suggested that the pooled effect were unlikely to be substantially altered (Fig. 10). As there were less than 10 included studies, publication bias analysis was not conducted.

### Adverse events

Three studies [16, 20, 21] mentioned adverse events during the intervention of physical activity. Among 951 participants, there were 2 knee pain, 1 shoulder pain, 1 back pain and 2 bone fractures which did not occur during exercise sessions reported. Overall, the incidence of adverse events was quite low, which suggesting that physical activity intervention was well-tolerated. However, only a few studies had reported on adverse events and the duration of trials ranged from 2 to 13 months. Therefore, the long-term safety of physical activity intervention needs further studies to prove.

## Discussion

In the present meta-analysis, we attempted to collect less biased evidence to identify the effect of physical activity intervention on patients with NAFLD. The included nine studies suggested that physical activity intervention can improvehepatic enzyme, most serum lipid and intra-hepatic lipid content in patients with NAFLD without diabetes. Although our meta-analysis results showed statistically significant effect, the magnitude of the improvement by physical activity interventions appears to be insignificant or small according to Cohen’s interpretation rule of thumb. This differs from previous reviews reporting that physical activity can significantly improve hepatic enzyme, serum lipid levels and intra-hepatic lipid content with a moderate to large pooled effect size [22, 23], but these previous studies included NAFLD patients with diabetes, so the improvement may partly due to antidiabetic medication or other treatment [23]. Besides, the pooled result showed physical activity had no significantly effect on glucose metabolism which may be also due to the population in our study was non-diabetic NAFLD patients.

The mechanism of physical activity intervention benefiting NAFLD patients is still unclear and there is no specific recommendation for types of physical activity. In the subgroup analyses of our study, we found compared with usual care without physical activity, resistance exercise alone can significantly improve hepatic enzyme and serum lipid, which is consistent with the results of systematic review by Kenneally et al. [24]. This finding is important for patients with NAFLD with obesity, for whom resistance exercise is more appropriate, as some types of aerobic exercise may result in joint stress or injury. However, our results suggested combination of aerobic and resistance exercise was less effective, only providing improvement for fasting insulin. This is probably due to the small sample size as the subgroup had only 76 participants in total. Longer exercise durations generally had better improvement effects, although the difference between subgroups was not significant. In the sensitivity analysis, results suggested pooled effect on hepatic enzyme and serum lipid parameters were robust. But the effect of physical activity on glucose metabolism parameters and intra-hepatic lipid content needs to be further ascertained by including more large RCTs, as only 4 studies had sufficient information on these parameters in our study. To the best of our knowledge, this study is currently the first systematic review that evaluated the comprehensive effectiveness of physical activity on hepatic enzyme, serum lipid, glucose metabolism and intra-hepatic lipid content in western and Chinese NAFLD patients without T2DM. The study population was restricted to subjects with NAFLD without T2DM.Although matching with an adequate control population can reduce the bias to some extents, the lack of information in anti-diabetic drug use of participants could still be a problem, considering different anti-diabetic medications have different effects on NAFLD [25]. We also excluded other factors that may have affected the meta-analysis results, such as diet adjustment or lipid-lowering medication.

Our study has several limitations. First, several of the included studies did not describe the intensity of the physical activity interventions clearly, therefore we could not perform subgroup analysis categorized by intensity, which may possibly influence the effect of the physical activity. Second, subgroup analysis according to different NAFLD diagnostic methods was inapplicable because Shojaee-Moradie et al. [19] used more than one diagnostic method for the confirmation of NAFLD. However, differences in the sensitivity and specificity of the different diagnostic methods could result in clinical heterogeneity. Biochemical tests have low specificity, and ultrasound is less accurate for patients with mild steatosis. The sensitivity of magnetic resonance imaging (MRI) for diagnosing hepatic steatosis is slightly higher than that of ultrasound. Nevertheless, all imaging examinations cannot detect the degree of liver inflammation and necrosis. Only liver biopsy can identify NAFL and NASH [26], among the non-invasive methods, ^1^H-MRS (MR spectroscopy) is the most accurate [27, 28]. Thirdly, there is a lack of information for histological improvement in the analyzed RCTs, which has been reported in several studies. Vilar-Gomez et al. conducted a RCT of 293 patients with histologically proven NASH and found degree of weight loss after a 52-week of diet was independently associated with improvements in all NASH-related histologic parameters (odds ratios = 1.1–2.0; *P* < 0.01, [29]). Another study also reported significant improvements in liver histology in NASH led by weight reduction achieved through 48 weeks lifestyle intervention including diet, exercise, and behavior modification among 31 overweight or obese individuals with biopsy-proven NASH [30]. Apart from that, the differing professional levels and clinical skills of the researchers in the included studies may also have affected the reported outcomes. In addition, among the included studies, the longest intervention duration was 48 weeks, therefore we could not determine the long-term benefit of physical activity. Another drawback is that although we searched the published studies thoroughly, we cannot rule out the possibility of missing unpublished studies with null effects, and we only included studies published in English and Chinese in this meta-analysis, which may explain the significant publication bias found for ALT.

## Conclusions

In conclusion, our meta-analysis suggests that regular physical activity, whether aerobic or resistance exercise alone, can improve hepatic enzyme, most serum lipid and intra-hepatic lipid in non-diabetic patients with NAFLD, but the effect size is generally small. Therefore, the expectation of great improvement in NAFLD from physical activity alone needs to be tempered with caution. Furthermore, future large-scale prospective RCTs are needed to determine the histological improvements, long-term benefits and safety of physical activity.

## Data Availability

The datasets used and/or analyzed during the current study available from the corresponding author on reasonable request.

## Abbreviations

ALT: Alanine aminotransferase
AST: Aspartate aminotransferase
CI: Confidence intervals
GGT: γ-glutamyl transferase
HDL-C: High-density lipoprotein cholesterol
LDL-C: Low-density lipoprotein cholesterol
NAFLD: Non-alcoholic fatty liver disease
NASH: Non-alcoholic steatohepatitis
RCT: Randomized controlled trial
SMD: Standardized mean difference
TC: Total cholesterol
TG: Triglycerides
